# Development of a Mitochondrial Targeting Lipid Nanoparticle Encapsulating Berberine

**DOI:** 10.3390/ijms24020903

**Published:** 2023-01-04

**Authors:** Ikuma Hori, Hideyoshi Harashima, Yuma Yamada

**Affiliations:** Faculty of Pharmaceutical Sciences, Hokkaido University, Kita-12, Nishi-6, Kita-ku, Sapporo 060-0812, Japan

**Keywords:** mitochondria, neuroblastoma, lipid nanoparticle, mitochondrial delivery

## Abstract

Delivering drugs to mitochondria, the main source of energy in neurons, can be a useful therapeutic strategy for the treatment of neurodegenerative diseases. Berberine (BBR), an isoquinoline alkaloid, acts on mitochondria and is involved in mechanisms associated with the normalization and regulation of intracellular metabolism. Therefore, BBR has attracted considerable interest as a possible therapeutic drug for neurodegenerative diseases. While BBR has been reported to act on mitochondria, there are few reports on the efficient delivery of BBR into mitochondria. This paper reports on the mitochondrial delivery of BBR using a lipid nanoparticle (LNP), a “MITO-Porter” that targets mitochondria, and its pharmacological action in Neuro2a cells, a model neuroblastoma. A MITO-Porter containing encapsulated BBR (MITO-Porter (BBR)) was prepared. Treatment with MITO-Porter (BBR) increased the amount of BBR that accumulated in mitochondria compared with a treatment with naked BBR. Treatment with MITO-Porter (BBR) resulted in increased ATP production in Neuro2a cells, which are important for maintaining life phenomena, compared with treatment with naked BBR. Treatment with MITO-Porter (BBR) also increased the level of expression of mitochondrial ubiquitin ligase (MITOL), which is involved in mitochondrial quality control. Our findings indicate that increasing the accumulation of BBR into mitochondria is important for inducing enhanced pharmacological actions. The use of this system has the potential for being important in terms of the regulation of the metabolic mechanism of mitochondria in nerve cells.

## 1. Introduction

Berberine (BBR), a plant metabolite that is classified as an isoquinoline alkaloid, exerts numerous pharmacological actions, including as an antioxidant and anti-inflammatory activity. Specifically, BBR has been reported to alleviate amyloid β-induced mitochondrial dysfunction [1]. Treatment with BBR also improved cognitive function by inhibiting the production of amyloid beta (Aβ) in a mouse model of Alzheimer’s disease (AD) [2] and short-term memory by inhibiting apoptosis in a mouse model of Parkinson’s disease (PD) [3]. Therefore, BBR has attracted interest as an active pharmaceutical drug for the treatment of neurodegenerative diseases including AD, PD, and Huntington disease [4,5,6]. 

BBR acts on the mitochondrial respiratory chain to induce a mitochondrial stress response, and its pharmacological action involves controlling the metabolic regulation [7]. BBR has been reported to exert a protective act on the central nervous system [8]. The accumulation of BBR into mitochondria in neurons is thought to have neuroprotective effects. However, our knowledge of whether the efficient delivery of BBR into mitochondria changes its physiological action remains quite limited. Therefore, we examined the issue of whether the use of a mitochondrial targeting lipid nanoparticle (LNP) to efficiently delivery BBR into mitochondria might be useful in terms of a treatment strategy.

We previously developed a MITO-Porter, an LNP for delivering cargoes to mitochondria. The MITO-Porter could be used to successfully deliver various cargoes including low-molecular-weight compounds and macromolecular compounds to a variety of targets [9,10,11,12]. This paper describes the encapsulation of BBR in a MITO-Porter that had been modified with octa-arginine (R8) (R8-MITO-Porter (BBR)). It has been reported that when the surface of an LNP is modified with R8, a membrane permeable peptide, this enhances its permeability to cells and targeting to mitochondria [9,13]. Moreover, R8-MITO-Porter can escape from macropinosomes to the cytosol, which prevents lysosomal degradation.

The goal of this study was to evaluate whether the delivery of BBR into mitochondria via a MITO-Porter system would result in an increase in the amount of BBR that accumulated in mitochondria. The successful use of this system would be expected to lead to enhanced functions of mitochondria due to the efficient delivery of BBR to mitochondria. In this study, we prepared an R8-MITO-Porter (BBR) and evaluated the physicochemical properties of the preparation. The accumulation of BBR in mitochondria was then measured, when naked BBR and the R8-MITO-Porter (BBR) with encapsulated BBR were added to cell cultures. The amount of intracellular ATP that was essential for cell survival was also measured. Finally, the level of expression of mitochondrial ubiquitin ligase (MITOL) that was involved in the quality control of mitochondria was measured. This pathway functioned to eliminate damaged mitochondrial proteins or parts of the mitochondrial network by mitophagy and to renew these components by producing the appropriate proteins and lipids through biogenesis, collectively resulting in mitochondrial turnover.

**Figure 1:**
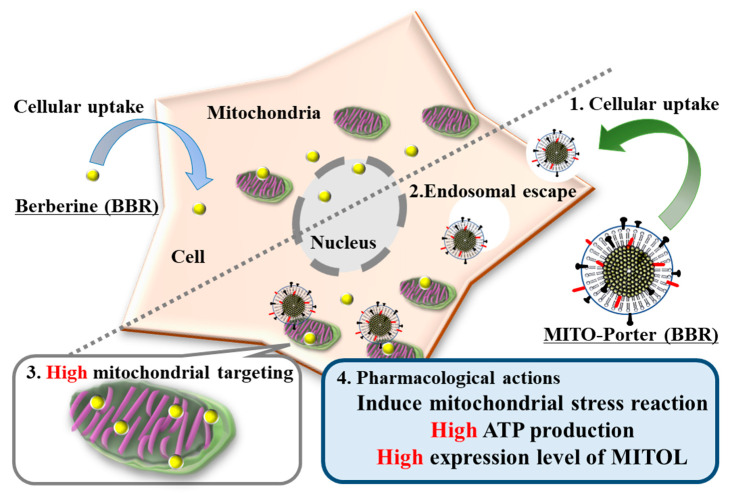
Schematic diagram showing the mitochondrial delivery of BBR using the MITO-Porter system. The use of the R8-MITO-Porter (BBR) resulted in an increased accumulation of BBR in mitochondria. This acted on the mitochondrial respiratory chain complex and induced a mitochondrial stress response. As a consequence, intracellular ATP production and MITOL expression were increased.

We hypothesized that the use of the R8-MITO-Porter (BBR) would result in an increase in the amount of BBR that accumulated into mitochondria compared with a treatment with naked BBR. As a consequence, a mitochondrial stress response was presumed to be effectively induced. This resulted in the production of increased amounts of ATP and the expression of MITOL (Figure 1). Increasing the accumulation of BBR in mitochondria via the MITO-Porter system was important for inducing enhanced pharmacological actions. Neuro2a cells, model neuroblastoma cells, were used as representative cells in these experiments. Neuro2a cells differentiate into neurons within a few days after the induction of differentiation and are considered to be a useful model for studying neuronal differentiation, axons, and signal transduction pathways.

## 2. Results

### 2.1. Preparation of the R8-MITO-Porter (BBR) Using the Lipid Film Hydration Method

The R8-MITO-Porter (BBR) was prepared by the lipid film hydration method. The resulting particles were composed of DOPE and SM (9:2 molar ratio). The physical properties of the prepared LNPs are summarized in Table 1. Our data showed that the R8-MITO-Porter was positively charged with a diameter of about 100 nm. Moreover, characteristics of the LNPs remained unchanged in culture medium (DOPE/SM-LNP: 78.6 ± 3.1, R8-MITO-Porter: 108 ± 4.2, R8-MITO-Porter (BBR): 107 ± 3.3). The recovery rate of BBR into the LNPs was about 10%.

The lipid compositions of prepared LNPs are DOPE and SM (9:2 molar ratio). Data are the mean ± S.D. (n = 3–9).

### 2.2. Evaluation of LNPs and BBR Uptake after Treatment with R8-MITO-Porter (BBR)

**Figure 2:**
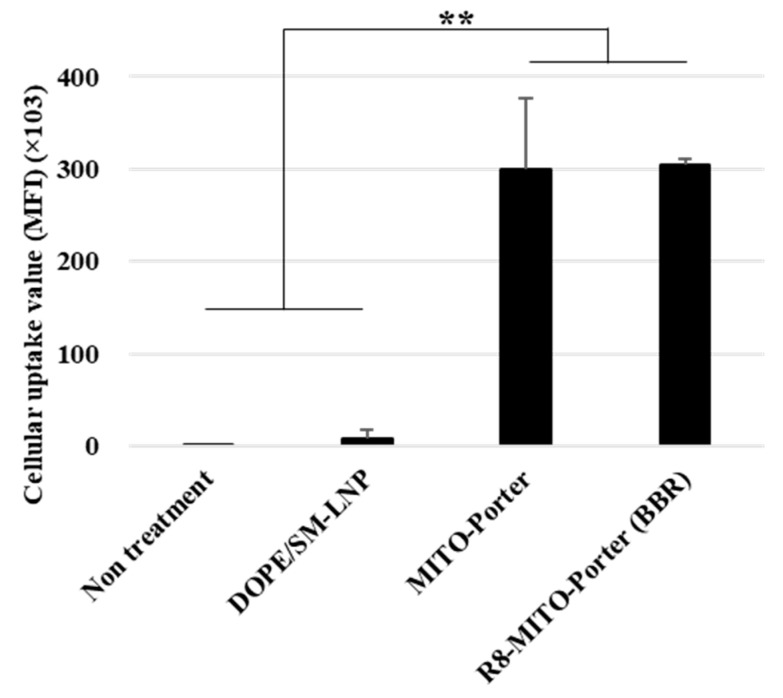
Evaluation of cellular uptake of LNPs. Cellular uptake of LNPs into Neuro2a cells was evaluated based on MFI using a FACS. The LNPs were labeled with DiD, a fluorescent dye, and the cells were analyzed after the LNP treatment. Data are represented as the mean ± S.D. (n = 3). ** *p* < 0.01, one-way ANOVA followed by Student–Newman–Keuls test.

The uptake of the LNPs into Neuro2a cells was evaluated by FACS analyses. After treating Neuro2a cells with the LNPs for 1 h, the Neuro2a cells were analyzed by CytoFLEX. The cellular uptake of the LNPs was expressed as the MFI. LNPs modified with R8 (R8-MITO-Porter and the R8-MITO-Porter (BBR)) showed a high cellular uptake efficiency. Nontreatment and the DOPE/SM-LNP were used as negative controls. The MFI for the R8-MITO-Porter was similar to that of the R8-MITO-Porter (BBR) (Figure 2). This result suggests that the uptake of the LNPs into Neuro2a cells was not affected by the presence of BBR.

**Figure 3:**
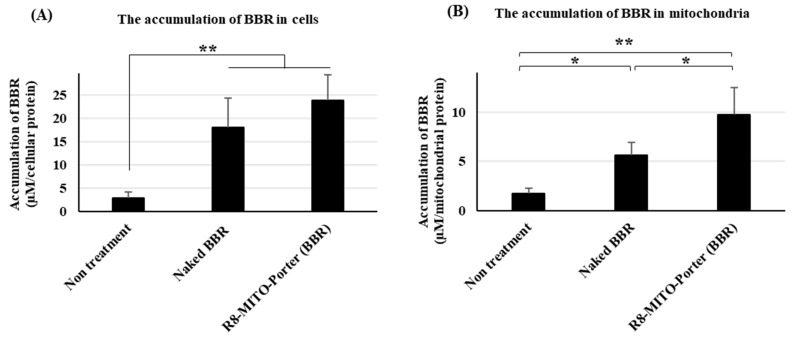
Evaluation of the accumulation of BBR into cells and mitochondria. After treatment with these samples, the medium was replaced, and the resulting preparation was incubated for 4 h. Measurement of the concentration of BBR in cells and mitochondria. (**A**) BBR concentration in cells. (**B**) BBR concentration in mitochondria. Data are represented as the mean ± S.D. (n = 3). * *p* < 0.05, ** *p* < 0.01, one-way ANOVA followed by Student–Newman–Keuls test.

Moreover, the accumulation of BBR in Neuro2a cells was measured after treatment with these samples. Nontreatment was used as a negative control. Treatment with R8-MITO-Porter (BBR) resulted in an increased accumulation of BBR into cells compared with the negative control. Treatment with naked BBR was similar to that for the R8-MITO-Porter (BBR) (Figure 3A). 

We then isolated mitochondria from Neuro2a cells and evaluated the accumulation of BBR into mitochondria. Treatment with naked BBR and R8-MITO-Porter (BBR) resulted in an increased accumulation of BBR in mitochondria compared with the negative control. It is also noteworthy that treatment with R8-MITO-Porter (BBR) caused more BBR to be accumulated into mitochondria compared with treatment with naked BBR (Figure 3B). This result suggests that BBR is efficiently delivered to mitochondria using the R8-MITO-Porter (BBR).

### 2.3. Evaluation of Mitochondrial Respiratory Function after Treatment with R8-MITO-Porter (BBR)

**Figure 4:**
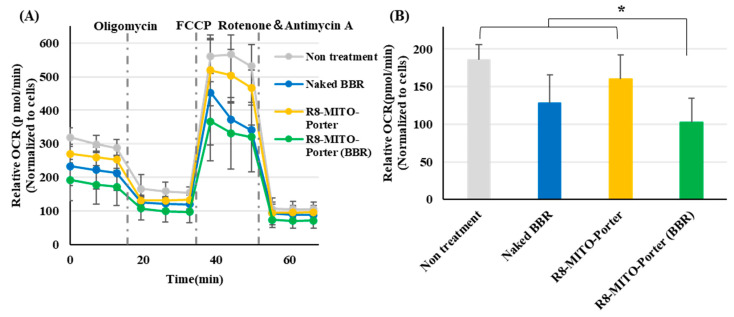
Evaluation of mitochondrial respiration function. Mitochondrial respiration function was evaluated using a SeahorseXFp analyzer at 4 h after treatment with these samples. (**A**) After the determination of OCR, the cells were sequentially treated with Oligomycin (1 µM), FCCP (2 µM), and rotenone plus antimycin A (0.5 µM). OCR was calculated by normalizing based on cell numbers. Data are represented as the mean ± S.D. (**B**) Basal OCR was calculated by normalizing using cell numbers. Data are represented as the mean ± S.D. (n = 4). * *p* < 0.05, one-way ANOVA followed by Student–Newman–Keuls test.

BBR acts on the mitochondrial respiratory chain complex I after being delivered to mitochondria. As a result, the basal oxygen consumption of mitochondria was decreased. The mitochondrial respiratory function was evaluated as the OCR, based on the Seahorse XF Analyzer assays. Nontreatment and the R8-MITO-Porter were used as negative controls. OCR changes in Neuro2a cells that had been treated with these samples represented basal respiration, maximal respiration, and pre-respiration (Figure 4A). Treatment with R8-MITO-Porter (BBR) caused a decrease in basal oxygen consumption compared with treatment with the negative controls (Figure 4B). This result suggests that the R8-MITO-Porter (BBR) was efficiently delivered into mitochondria and that it clearly had an effect on the respiratory chain complex.

### 2.4. Evaluation of Intracellular ATP Amounts after Treatment with R8-MITO-Porter (BBR)

**Figure 5:**
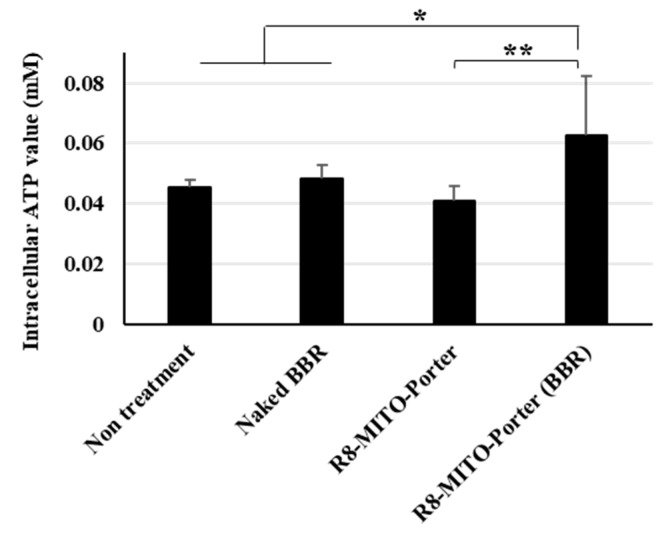
Evaluation of intracellular ATP production. The amount of intracellular ATP was evaluated using an ATP assay kit at 4 h after treatment with these samples. Data are represented as the mean ± S.D. (n = 6). * *p* < 0.05, ** *p* < 0.01, one-way ANOVA followed by Student–Newman–Keuls test.

We hypothesized that inducing a mitochondrial stress response by altering mitochondrial respiratory function might improve mitochondrial function. The amount of intracellular ATP required for life support in nerve cells was evaluated using an ATP assay kit. Nontreatment and the R8-MITO-Porter were used as negative controls. Treatment with R8-MITO-Porter (BBR) caused an increase in intracellular ATP levels compared with the treatment with the negative controls (Figure 5). Moreover, treatment with R8-MITO-Porter (BBR) increased the intracellular ATP levels to a greater extent compared with treatment with naked BBR (Figure 5). This result suggested that mitochondrial function was improved, and the levels of intracellular ATP were increased after the induction of the mitochondrial stress response by treatment with R8-MITO-Porter (BBR).

### 2.5. Evaluation of MITOL Expression Level after Treatment with R8-MITO-Porter (BBR) 

**Figure 6:**
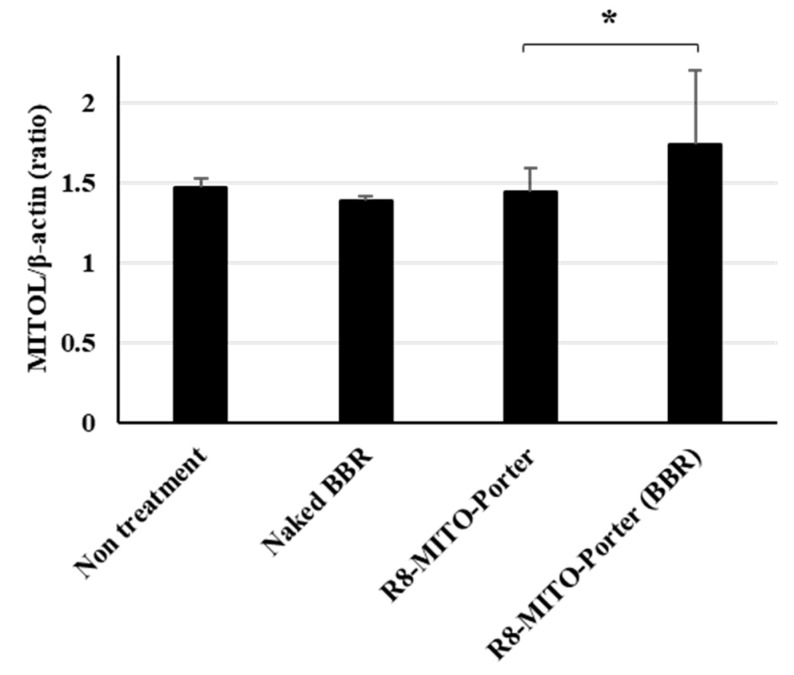
Evaluation of expression level of MITOL. Expression level of MITOL was evaluated using quantitative real-time PCR at 24 h after treatment with these samples. Fold change gene expression was determined using Ct values and normalized to β-action expression. Data are represented as the mean ± S.D. (n = 4). * *p* < 0.05, one-way ANOVA followed by Student–Newman–Keuls test.

It has been reported that the intracellular ATP concentration activated the ubiquitin proteasome system [14]. Treatment with R8-MITO-Porter (BBR) caused increased levels of intracellular ATP. As a result, the gene expression level of MITOL was presumed to increase. The gene expression level of MITOL was evaluated by quantitative PCR. Nontreatment and R8-MITO-Porter were used as negative controls. The relative gene expression level was also calculated by normalizing with β-actin. Treatment with R8-MITO-Porter (BBR) caused an increased gene expression of MITOL compared with treatment with R8-MITO-Porter (Figure 6). This result suggested that mitochondrial quality control was upregulated with increased MITOL gene expression by treatment with R8-MITO-Porter (BBR).

### 2.6. Evaluation of Cell Viability after Treatment with R8-MITO-Porter (BBR)

**Figure 7:**
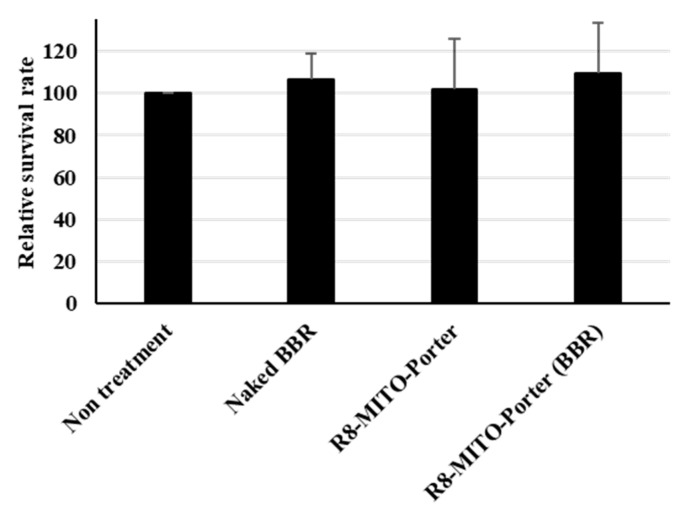
Evaluation of cell viability. After treatment with these samples, the medium was replaced, and the resulting suspension was incubated for 4 h. Cell viability was evaluated using PremixWST-1 reagent. Relative cell viability was calculated by normalizing the cell viability for the nontreatment. Data are represented as the mean ± S.D. (n = 3). One-way ANOVA followed by Student–Newman–Keuls test. No significant difference was detected.

Cell viability was evaluated by the WST-1 reagent based on mitochondrial enzyme activity after treatment with these samples. Nontreatment and the R8-MITO-Porter were used as negative controls. The value for treatment with R8-MITO-Porter (BBR) was similar to that for the other samples (Figure 7). An evaluation of cell viability indicated that treatment with R8-MITO-Porter (BBR) was not highly toxic to the cells.

## 3. Discussion

This paper focuses on the issue of whether the R8-MITO-Porter (BBR) could be used for the efficient delivery of BBR to mitochondria. Treatment with R8-MITO-Porter (BBR) was clearly found to increase the accumulation of BBR into Neuro2a cells (Figure 3). 

It is generally thought that intracellular BBR is largely localized in mitochondria, the nucleus, and the cytoplasm depending on the concentration of BBR [15,16]. Efficient delivery of BBR to mitochondria is very important in terms of enhancing its pharmacological action. As shown in Figure 3, treatment with R8-MITO-Porter (BBR) caused an increased accumulation of BBR into Neuro2a cells compared with treatment with the other samples. This result was consistent with previous reports of increased drug delivery via cellular uptake and mitochondrial membrane fusion using a MITO-Porter with R8 on the surface [9,17]. As a consequence of the induced mitochondrial stress response, the amount of the intracellular ATP increased after treatment with R8-MITO-Porter (BBR). The efficient delivery of BBR into mitochondria, the main site of action, by this system would be expected to lead to enhanced pharmacological actions.

As shown in Figure 4, treatment with R8-MITO-Porter (BBR) had a dramatic impact on mitochondrial respiratory function compared with treatment with the other samples that were examined. An increase in ADP due to a temporary decrease in respiratory activity stimulated the action of ATP synthase and changed the mitochondrial respiratory function. It has been reported that mitochondrial respiration was decreased when all of the ADP was converted into ATP [18]. It has also been reported that ATP production was increased in isolated mitochondria under conditions of hypoxia [19]. As shown in Figure 5, a temporary decrease in respiratory activity by treatment with R8-MITO-Porter (BBR) resulted in an increase in the levels of intracellular ATP. Our results suggest that the temporary increase in the conversion of ADP to ATP and the resulting increased ATP levels would decrease basal oxygen consumption. It has been reported that an increase in ADP activated AMP-activated protein kinase (AMPK), which results in an improved intracellular quality control and enhanced cell function [20,21]. Our results supported the previously reported idea that BBR acts as a guardian of the mitochondria.

As shown in Figure 6, treatment with the R8-MITO-Porter (BBR) caused in increase in the level of expression of MITOL that was involved in mitochondrial quality control. It has been reported that MITOL alleviates the pathology of Alzheimer’s disease via blocking generation of the amyloid-β oligomer [22] and is expected to be a potential drug discovery target for the treatment of neurodegenerative diseases [23]. Therefore, an increase in intracellular ATP production as well as the level of MITOL expression by R8-MITO-Porter (BBR) would be expected to serve as a therapeutic base technology for the treatment of neurodegenerative diseases.

BBR has also been reported to exert a protective function on neurons [21,24]. The induction of a response to mitochondrial stress by BBR has been reported to be important for developing enhanced neuroprotection [7,25,26]. Based on the above findings, we concluded that the use of the R8-MITO-Porter (BBR) led to enhancements in pharmacological action due to more efficient delivery of BBR into mitochondria. The use of the R8-MITO-Porter (BBR) is expected to lead to enhanced pharmacological actions by inducing a more effective mitochondrial stress response. Previously, we have shown the successful delivery of cationic LNPs (R8-MITO-Porter) to the brain [27]. Moreover, treatment with cationic NPs that contain encapsulated BBR has been reported to improve learning and memory function at lower doses than treatment with naked BBR [28]. This study confirms that the administration of BBR-NPs, at ~1/6 of the therapeutic recommended dose of BBR (7 mg/kg compared with 50 mg/kg), significantly improved learning and memory function. The characteristics of these NPs were similar to that for the R8-MITO-Porter. Therefore, it was presumed that it achieved a pharmacological effect due to administration of MITO-Porter at the same dose. These collective findings suggest that the R8-MITO-Porter (BBR) has the potential for use as an innovative therapeutic strategy for the treatment of neurodegenerative disease. However, the limitation of this study was that pharmacological actions of BBR against Neuro2a cells were examined only in vitro. These experiments need to be repeated using another cell line (PC12 cell, SHSY5Y cell, and primary neurons). Moreover, we need to further verify the in vivo experimental results, in order to make the treatment for neurodegenerative disease more practical. Further studies will involve the direct evaluation of the physicochemical characterizations of the R8-MITO-Porter (BBR) and the amount of activated AMPK that is produced in an in vivo validation study.

## 4. Materials and Methods

### 4.1. Materials

The 1,2-dioleoyl-sn-glycero-3-phoshoetanolamine (DOPE) and methoxy polyethene glycol 2000 (DMG-PEG-2k) were obtained from the NOF Corporation (Tokyo, Japan). Sphingomyelin (SM) was obtained from Avanti Polar Lipids (Alabaster, AL, USA). Stearylated R8 (STR-R8) was obtained from TORAY Research Center, Inc. (Tokyo, Japan). The 1,1′-Dioctadecyl-3,3,3′,3′-Tetramethylindodicarbocyanine, 4-Chlorobenzenesulfonate (DiD) and the Mitochondria Isolation Kit for cultured cells were obtained from Thermo Fisher Scientific Inc. (Waltham, MA, USA). All other chemicals that were used were commercially available, reagent-grade products.

### 4.2. Preparation of the R8-MITO-Porter (BBR)

The MITO-Porter was prepared by the lipid film hydration method. In a glass tube, a total of 900 nmol of lipids (DOPE/SM = 9/2, molar ratio) was dissolved in an organic solvent (ethanol/chloroform = 1/1, per volume). A thin lipid film was formed by the evaporation of organic solvents over a period of more than 2 h, after which 10 mM HEPES buffer (pH 7.4) containing BBR (600 µM) was added, followed by sonication for 1 min in a bath-type sonicator. Finally, to attach the R8 to the surface of the LNP, a solution of STR-R8 (10 mol% of total lipids) was added to the resulting suspension, followed by incubation for 30 min at room temperature. The properties of the resulting LNPs were measured by a Zetasizer Nano ZS (Malvern Instruments, Worcestershire, UK). The lipid compositions of these LNPs are summarized in Table 1.

### 4.3. The Method of Removed Unencapsulated BBR

The unencapsulated BBR was removed by dialysis, and the resulting LNPs were processed by an ultrafiltration method. Spectra/Por 4-dialysis membranes (MWCO 12,000–14,000) were obtained from IEDA TRADING Co. (Tokyo, Japan). The Amicon ultra-15 100K was obtained from Merck Millipore Ltd. (Darmstadt, Germany). In the dialysis method, the LNP solution was transferred to a dialysis membrane and rotated at 700 rpm, 25 °C, and 2 h in 10 mM HEPES buffer (pH 7.4). In the ultrafiltration method, the LNP solution was transferred to an Amicon ultra-15 100K and centrifuged at 1000 × *g*, 25 °C, and 40 min in 10 mM HEPES buffer (pH 7.4). The absorbance of berberine at 350 nm was measured by an absorption spectrometer. The recovery rate of BBR was calculated as follows:

Recovery rate (%) = (Final concentration of BBR/First concentration of BBR) × 100

### 4.4. Cell Cultures and Transfection Study

Neuro2a cells (CCL-131), a mouse neuroblastoma cell line, were obtained from the ATCC (Manassas, VA, USA). Eagle’s Minimum Essential Medium (EMEM) was obtained from the ATCC. Fetal bovine serum (FBS), penicillin, and streptomycin were obtained from Sigma-Aldrich (St. Louis, MO, USA). The cells were maintained in complete medium, which is EMEM medium supplemented with 10% FBS, penicillin (100 U/mL), and streptomycin (100 µg/mL). The cells were cultured under an atmosphere of 5% CO_2_/air at 37 °C. One day prior to treatment with the samples, Neuro2a cells were seeded on plates or dishes for each experiment. Transfection was performed by the following method. Cells were washed with phosphate buffered saline without calcium chloride (PBS (-)), the media were replaced with the LNPs (final lipid concentration: 0.36 µM) containing EMEM without serum, and the resulting suspension was then incubated for 30 min under an atmosphere of 5% CO_2_/air at 37 °C.

### 4.5. Evaluation of Cellular Uptake by a Fluorescence Activated Cell Sorter (FACS)

Neuro2a cells were cultured in complete medium for 24 h under an atmosphere of 5% CO_2_/air at 37 °C. DiD (0.5 mol% of total lipids) was integrated into lipid membranes and used as an indicator of the cellular uptake of the particles. DiD, a carbocyanine dye, was retained on the particle membrane as the result of hydrophobic interactions during particle formation. Cells were transfected with the LNPs. After replacement with complete medium and incubation for 30 min under an atmosphere of 5% CO_2_/air at 37 °C, the cells were washed twice with PBS (−) containing heparin (20 U/mL) and then collected with trypsin 0.25% EDTA. After centrifugation at 700× *g*, at 4 °C for 3 min, the supernatant was removed, and the collected cells were suspended in FACS buffer, which contained bovine serum albumin (5 mg/mL) and sodium azide (1 mg/mL) in PBS (−). After filtration through a nylon mesh, the cells were analyzed by means of a CytoFLEX Flow Cytometer (Backman Coulter Inc., Brea, CA, USA). DiD was excited by a 638 nm light, and the band pass filter for the fluorescence detection was set to 660 nm. The value for the cellular uptake of each LNP was expressed as the mean fluorescent intensity (MFI), the integrated fluorescence intensity, and the cell counts.

### 4.6. Evaluation of the Accumulation of BBR in Cells and Isolated Mitochondria

Neuro2a cells were cultured in complete medium for 72 h under an atmosphere of 5% CO_2_/air at 37 °C. Cells were transfected with the LNPs. The medium was then replaced with a complete medium and incubated for 30 min under an atmosphere of 5% CO_2_/air at 37 °C. The cells were washed twice with PBS (−) containing heparin (20 U/mL) and then collected with a cell scraper. A Mitochondria Isolation Kit for Cultured Cells (Thermo Fisher Scientific Inc.) was used to isolate mitochondria from the cells according to the manufacturer’s manual. The cells or isolated mitochondria were placed in a solvent (Tris buffer/SDS = 1/10, per volume). The concentrations of protein (280 nm) and BBR (350 nm) in the solubilized cells and mitochondria were measured using a NanoDrop One^C^ (ND-ONEC-W, Thermo Fisher Scientific Inc.). The BBR concentration was calculated by normalizing with the protein concentration.

### 4.7. Evaluation of Mitochondrial Respiratory Function

Mitochondrial respiratory function was evaluated using a Seahorse XF HS Mini Analyzer (Agilent Technologies Inc., Santa Clara, CA, USA). Neuro2a cells were cultured in complete medium for 24 h under an atmosphere of 5% CO_2_/air at 37 °C. The cells were transfected with LNPs. It was then replaced with complete medium and incubated for 3.5 h under an atmosphere of 5% CO_2_/air at 37 °C. The medium was replaced with the Seahorse XF assay culture medium (Agilent Technologies Inc.), which contained glucose, pyruvate, and glutamine, and the plate was incubated for 1 h at 37 °C in the absence of CO_2_.

Respiratory capacity was measured using the Seahorse XF cell mito stress test kit (Agilent Technologies Inc.). After the determination of basal oxygen consumption rates (OCRs), the cells were sequentially treated with Oligomycin A (2 µM), FCCP (1.5 µM), and rotenone/antimycin A (0.5 µM). Viable cell numbers were counted and used to normalize the OCR.

### 4.8. Evaluation of Intracellular ATP Production

Total ATP production was measured through a colorimetric/fluorometric ATP Assay Kit (Abcam, Cambridge, UK) according to the manufacturer’s manual. Neuro2a cells were cultured in complete medium for 24 h under an atmosphere of 5% CO_2_/air at 37 °C. The cells were then transfected with LNPs. After replacing the medium with complete medium and incubation for 3.5 h under an atmosphere of 5% CO_2_/air at 37 °C, the cells were collected, washed with PBS, and then resuspended in ATP assay buffer. The cells were homogenized and then centrifuged at 13,000 × *g* at 4 °C for 5 min to remove any insoluble material. The supernatants were then collected and incubated with the ATP probe and the reaction mix for 30 min. Absorbance was detected at 535/587 nm using a microplate reader (EnSpire^®^ Multimode PlateReader, Perkin Elmer, Waltham, MA, USA). The sample concentration was calculated by the calibration curve of the ATP standard solution.

### 4.9. Evaluation of MITOL Gene Expression Level

Neuro2a cells were cultured in complete medium for 24 h under an atmosphere of 5% CO_2_/air at 37 °C. The cells were transfected with the LNPs. It was then replaced with complete medium and incubated for 1 day under an atmosphere of 5% CO_2_/air at 37 °C. The cells were washed twice with PBS (−) containing heparin (20 U/mL) and then collected with trypsin 0.25% EDTA. After centrifugation at 700× *g* at 4 °C for 3 min, the supernatant was removed. The collected cells were suspended in RLT buffer, and RNA was extracted using an RNeasy mini kit (Qiagen, Hilden, Germany) according to the manufacturer’s manual. The extracted RNA was purified by a TURBO DNA-free kit (Thermo Fisher Scientific Inc.) according to the manufacturer’s manual. The purified RNA was reverse transcribed into cDNA using a High-Capacity RNA to cDNA kit (Thermo Fisher Scientific Inc.). Quantitative real-time PCR was performed using a THUNDERBIRD SYBR qPCR Mix (TOYOBO, Osaka, Japan). The amplification conditions consisted of one cycle at 95 °C, 1 min, and were followed by 40 cycles at 95 °C, 15 s, and at 60 °C, 1 min. Fold change gene expression was determined using cycle threshold (Ct) values and normalized to β-action expression. The primers are listed below.

MITOL:

forward: 5′–GAG CAG TGA CAG TGA TGC AG–3′, 

reverse: 5′–AGG ACA ACC TAT CCC TGG AA–3′

β-actin: 

forward: 5′–AGA GGG AAA TCG TGC GTG AC–3′, 

reverse: 5′–CAA TAG TGA TGA CCT GGC CGT–3′

### 4.10. Evaluation of Cell Viability 

Neuro2a cells were cultured in complete medium for 24 h under an atmosphere of 5% CO_2_/air at 37 °C. Cells were transfected with the LNPs. It was then replaced with complete medium and incubated for 1 h under an atmosphere of 5% CO_2_/air at 37 °C. The medium was replaced with complete medium containing PremixWST-1 reagent (TAKARA Bio Inc., Shiga, Japan) and incubated for 2 h under an atmosphere of 5% CO_2_/air at 37 °C. Absorbance was detected at 440 and 650 nm using a microplate reader. Relative cell viability was calculated by normalizing the cell viability of the control.

Cell viability (%) = Vt/Vu × 100

where Vt and Vu represent the cell viability for treated and untreated cells with samples, respectively.

### 4.11. Statistical Analysis 

Data are expressed as the mean ± SD for the indicated number of experiments. We used the Excel Statistical Program File (ystat2013) as the statistical software. For multiple comparisons, one-way ANOVA was performed, followed by the Student–Newman–Keuls test. Levels of *p* < 0.05 were considered to be significant.

## Figures and Tables

**Table 1:** Characteristics of the LNPs used in this study.

LNP Type	Diameters (nm)	Polydispersity Index (PdI)	ζ-Potential (mV)	Recovery Rate (%)
DOPE/SM-LNP	102 ± 6.6	0.21 ± 0.06	−5.36 ± 2.4	
R8-MITO-Porter	97.6 ± 5.9	0.26 ± 0.02	29.5 ± 3.7	
R8-MITO-Porter (BBR)	93.6 ± 1.6	0.28 ± 0.01	30.1 ± 4.8	12.2 ± 4.6

## Data Availability

The datasets used and/or analyzed during the current study available from the corresponding author on reasonable request.
